# Predicting Residual Astigmatism in Cataract Surgery

**DOI:** 10.3390/vision6040070

**Published:** 2022-11-24

**Authors:** Atsushi Kawahara

**Affiliations:** Yoshida Eye Hospital, Hakodate 041-0851, Japan; atsusi-k@coral.plala.or.jp

**Keywords:** astigmatism, cataract surgery, toric intraocular lens (IOL), residual astigmatism, with-the-rule (WTR) astigmatism, against-the-rule (ATR) astigmatism, oblique astigmatism, corneal astigmatism, refractive astigmatism, vector analysis

## Abstract

The purpose of this review is to evaluate the prediction of postoperative residual astigmatism and to determine the best prediction method for astigmatism correction. In recent findings for residual astigmatism in non-toric monofocal intraocular lens (IOL) implanted eyes, vector analysis can be used to correctly evaluate residual astigmatism by decomposing it. In predicting residual astigmatism, the with-the-rule (WTR) and against-the-rule (ATR) astigmatism components can now be almost predicted. This may be due to advances in inspection equipment and surgical technique. However, there are still issues with the oblique astigmatism component. In addition, corneal astigmatism is the most important predictor of postoperative residual astigmatism, and other predictors, such as refractive astigmatism, age, and lens thickness, have also been mentioned. However, all but corneal astigmatism are questionable because of the possibility of confounding variables. Total corneal astigmatism is more accurate in predicting residual astigmatism than anterior corneal astigmatism. Several predictions of residual astigmatism have been reported, but complete prediction has not been possible. Further research is needed, especially in predicting oblique astigmatism. However, I emphasize that the accuracy of predicting WTR and ATR astigmatism has improved considerably and can be predicted using regression equations with total corneal astigmatism.

## 1. Introduction

Residual astigmatism after cataract surgery is a common refractive error observed in a significant percentage of patients [1]. Residual astigmatism of 0.5 diopters or greater decreases uncorrected visual function and patient satisfaction [2,3]. Corneal astigmatism is a major contributor to residual astigmatism, but other contributions include any posterior corneal surface effects and other contributions, such as physiologic intraocular lens (IOL) tilt or decentration, refractive changes in the anterior and posterior corneal surfaces from the cataract incisions, and systematic differences in measured keratometric versus actual corneal refractive astigmatism [4,5]. 

The efficacy and usefulness of toric IOL implantation to reduce existing astigmatism during cataract surgery is well documented [6,7,8]. Although the principle of toric IOLs is to eliminate ocular astigmatism by neutralizing corneal astigmatism, it is often experienced that residual astigmatism can occur even with accurate implantation [9]. Therefore, it is important to predict postoperative ocular astigmatism (refractive astigmatism) before surgery. However, there are many reports that analyzed the results of residual astigmatism after toric IOL implantation to predict postoperative astigmatism, but errors in the toric IOL calculation formula and the orientation of IOL placement may have biased the results [10]. In order to discuss postoperative residual astigmatism, it is considered desirable to analyze residual astigmatism after implantation of a non-toric IOL.

The purpose of this study was to evaluate the predictors and predictive accuracy of residual astigmatism after cataract surgery.

## 2. Analysis Method for Astigmatism

Astigmatism has a cylindrical refractive power and axis that is best described mathematically by a vector. Vectors allow combinations of magnitude and direction to be represented in a single mathematical expression [11]. To evaluate dynamic changes in astigmatism, it is not sufficient to analyze data separately for the magnitude and axis of astigmatism, because the statistical analysis of angular data (astigmatic axis, compass orientation, etc.) is fundamentally different from the analysis of non-directional data [12]. The dynamic nature of astigmatism has been studied primarily using graphical vector analysis [13], polar value method [14], power vector analysis [12], and X-Y coordinate analysis [15,16]. In recent years, power vector analysis and X-Y coordinate analysis have become mainstream.

The power vector analysis decomposes astigmatism into vertical/horizontal astigmatism components and oblique astigmatism components. The vertical/horizontal astigmatism component is represented as Jackson cross cylinder, axes 90 and 180 degrees (J0), and the oblique astigmatism component as Jackson cross cylinder, axes 45 and 135 degrees (J45). A positive J0 indicates with-the-rule (WTR) astigmatism, and a negative J0 indicates against-the-rule (ATR) astigmatism; a positive J45 indicates counterclockwise oblique astigmatism, and a negative J45 indicates clockwise oblique astigmatism. This concept of independence formalized in the mathematical orthogonality concepts of J0 and J45 simplifies the practical issues involved in the combination, comparison, and statistical analysis of spherocylindrical lenses or refraction errors [8,12].

The X–Y coordinate analysis decomposes the astigmatism into vertical/horizontal (WTR and ATR) and oblique astigmatism components. For this analysis, the vertical/horizontal astigmatism component is defined as X and the oblique astigmatism component as Y. Negative values of X indicate ATR astigmatism, and positive values indicate WTR astigmatism. As with power vector analysis, the concepts of X and Y allow for mathematical and statistical analysis [15,16,17].

## 3. Prediction of Residual Astigmatism

Refractive astigmatism includes a corneal and an intraocular astigmatism component [18]. The main component of intraocular astigmatism is lens astigmatism. It has been believed that there is a statistical linear relationship between refractive astigmatism and corneal astigmatism. Javal proposed that refractive astigmatism can be calculated from corneal curvature when the major meridians of the eye are 0 and 90 degrees (refractive astigmatism = 1.25 × corneal (keratometric) astigmatism + (−0.50 × 90°)) [19]. Javal assumed a constant related to intraocular astigmatism of −0.50 diopters, but this is an average estimate for the population because of individual differences in the lens component. This method of calculating refractive astigmatism is known as Javal’s rule. Subsequently, refractive and corneal astigmatism were considered to have a nonlinear relationship [20].

Table 1 summarizes reports that investigated the correlation between refractive and corneal astigmatism in pseudophakic eyes receiving non-toric IOLs using vector analysis. In all of them, linear regression analysis was performed. The coefficients of determination in the reports of Teus et al. [21] and Tejedor et al. [22] were less than 0.5. Although it is not surprising that the correlation is lower than in multiple regression analysis because they were calculated by single regression analysis, it must be said that the correlation between refractive and corneal astigmatism is low from this result. In the study of Leffler et al. [23], the coefficient of determination was low, despite multiple regression analysis. The population included cases operated on by a resident, cases in which the IOL was fixed in the sulcus, and cases in which the incision was sutured, which included multiple factors that would result in a low correlation. High correlations were observed in reports other than those mentioned above [24,25,26]. This may be due to the evolution of cataract surgery techniques and testing equipment in recent years, although it may also be due to the influence of multiple regression analysis. However, in a previous study [25], the coefficient of determination of the WTR/ATR astigmatism component was relatively low. The significant difference from other reports [24,26] may be due to the surgeon and the associated degree of surgical invasiveness. A recent study [26] improved the coefficient of determination of the WTR/ATR astigmatism component to an approximation of 1. With the improvement of cataract surgery practice, the WTR/ATR component has become almost predictable. On the other hand, the coefficient of determination for the oblique astigmatism component remained around 0.7. Linear regression analysis may have limitations in predicting oblique astigmatism; Abulafia et al. [27] presented a regression equation with a coefficient of determination of 0.96 in their analysis of linear regression for oblique astigmatism. In this report, the analysis was based on residual astigmatism in toric IOL implanted eyes, but the reason for such a high accuracy in predicting oblique astigmatism is unclear. In addition to corneal astigmatism, refractive astigmatism, age, and lens thickness were selected as explanatory variables in the multiple regression analysis. The standard partial regression coefficient, which indicates the importance of each explanatory variable in the regression equation, was highest for corneal astigmatism in all reports. The standard partial regression coefficients other than corneal astigmatism are approximately 1/4 to 1/3 of those for corneal astigmatism [24,25,26], indicating that those explanatory variables are not highly important. They also have the potential for confounding. It is widely known that corneal astigmatism is the major contributor to refractive astigmatism. Corneal astigmatism has been shown to change to ATR astigmatism with age [28,29,30,31,32], and lens thickness is correlated with age [23,33]. These results suggest that multiple regression analysis that includes corneal astigmatism as an explanatory variable has limitations. In the future, it may be better to use the total surgically induced astigmatism analysis [34] to investigate predictors other than corneal astigmatism.

## 4. Factors Affecting Prediction Accuracy

The most important predictor of residual astigmatism remains corneal astigmatism. The magnitude of change in corneal astigmatism depends on the invasiveness of the surgery. Table 2 summarizes the corneal astigmatism examination and surgical technique used in each of the reports listed in Table 1.

### 4.1. Corneal Astigmatism Examination

Corneal curvature measurement using a keratometer has long been used to measure corneal astigmatism. The keratometer measures the frontal curvature of the cornea at several points in the center of the front surface of the cornea and calculates the total corneal keratometric reading (K value) by decreasing the refractive index. This calculation uses the keratometric index to estimate the refractive power of the total cornea from the measured anterior corneal curvature. That is, the K value is not the actual measured value, but an estimated average corneal refractive power that takes into account the anterior surface of the cornea, the various layers, and the negative refractive power of the posterior surface of the cornea. Therefore, astigmatism values calculated from K values do not accurately represent the true total corneal astigmatism, which is the actual measurement of anterior and posterior corneal curvature [35,36,37]. Therefore, it has been reported that it is essential to include measurement of posterior corneal astigmatism for accurate astigmatism prediction after cataract surgery [38,39]. However, consideration of Table 1 and Table 2 shows that the use of total corneal astigmatism does not necessarily increase the correlation. Despite the fact that previous studies [22,25] assessed residual astigmatism by total corneal astigmatism, the correlation was lower than that of keratometry when the surgical technique was nearly similar. These reports used a Scheimpflug camera, and Tejedor et al. [22] concluded that this was due to the fact that corneal curvature measurements with a Scheimpflug camera are relatively less reproducible than refractive values with an autokeratometer [40,41]. On the other hand, it has been reported that the Scheimpflug camera showed high repeatability with respect to measurement accuracy [42]. The correlation was good when total corneal astigmatism measured by swept-source anterior segment optical coherence tomography (OCT) was used. Because anterior segment OCT measures the anterior and posterior corneal curvature based on a single principle, the measurements are more accurate than those of other systems [43]. Measurements obtained with anterior segment OCT have excellent repeatability and reproducibility [44]. Residual astigmatism prediction using total corneal astigmatism is preferable, but its measurement equipment may have to be considered. The principle of the measurement equipment may also need to be modified. The surface topography of the anterior surface of the human cornea is an ellipsoid, not part of the sphere of a cone. Topographic information, and algorithms based on it, may lead to erroneous results. This, of course, also affects the accuracy of the prediction. Further study is needed here as well.

### 4.2. Surgical Technique

Recent improvements in cataract surgery techniques have reduced surgical invasiveness. The incision width has decreased, with the standard width being less than 2.5 mm, which has led to fewer changes in corneal shape and earlier stabilization [34,45,46]. In addition, because side ports affect corneal shape by rotating the axis of astigmatism [47], fewer side ports are better for predicting residual astigmatism. With regard to IOLs, one-piece IOLs are currently the most common; compared to three-piece IOLs, one-piece IOLs are more stable with less IOL tilt and dislocation [48,49,50]. The stability of the IOL position affects postoperative refraction [48]. In this study, too, the correlation was better for reports with main incisions smaller than 2.5 mm than for other reports, and in addition, the number of side ports seemed to have an effect. For the report by Leffler et al. [23], in addition to the factors presented in the previous section, the inclusion of some cases with main incision wounds larger than 3 mm may have reduced the correlation.

### 4.3. Dry Eye

Dry eye increases corneal irregular astigmatism and higher order aberrations, resulting in poor visual function [51,52]. In particular, unstable tear fluid has been reported to alter the magnitude and axis of astigmatism [53,54]. It is well known that cataract surgery can cause dry eye. It has also been shown that preoperative dry eye is often missed [55], and clinicians should be aware of the perioperative ocular surface examination, as dry eye can have a negative impact on prediction accuracy. In the reports reviewed in this study, dry eye evaluations were not performed on the subjects.

## 5. Conclusions

Prediction of postoperative residual astigmatism is not a simple matter because, in cataract surgery, an incision is made in the cornea (or sclera), and the lens is converted to an IOL, which adds surgically induced ocular astigmatism to the preoperative astigmatism. The only definitive predictor of residual astigmatism is and always has been corneal astigmatism, but prediction using total corneal astigmatism measured by anterior segment OCT is preferable. In addition, preoperative dry eye evaluation is essential to ensure accuracy of prediction.

Recent technological advances have made it almost possible to predict WTR/ATR astigmatism by using regression equations, but the prediction of oblique astigmatism is still incomplete and requires further study. In another study, the Baylor toric IOL nomogram [56] was proposed as a method to determine the corrected astigmatism power in toric IOLs, but this method is also limited in its application to eyes with WTR and ATR astigmatism only and cannot be used for eyes with oblique astigmatism.

At present, it is best to perform a preoperative vector analysis of astigmatism and predict residual astigmatism by regression equation when there is little or no oblique astigmatism component. Since regression equations are likely to vary by surgeon and measurement equipment, it is recommended that a personal regression equation be prepared.

## Figures and Tables

**Table 1:** Overview of studies focusing on the correlation between refractive and corneal astigmatism using vector analysis in eyes receiving non-toric intraocular lenses (IOLs).

Author	Regression Analysis	*R*^2^ Value		Predictor
		WTR/ATR Astigmatism Component	Oblique Astigmatism Component	
Teus et al. 2010 [21]	Univariate	0.29	0.36	
Tejedor et al. 2013 [22]	Univariate	0.49 * 0.13	0.42 * 0.20	
Leffler et al. 2008 [23]	Multivariate	0.51	0.17	Corneal and refractive astigmatism
Kawahara et al. 2017 [24]	Multivariate	0.85	0.70	Corneal and refractive astigmatism
Kawahara et al. 2020 [25]	Multivariate	0.55	0.63	Corneal astigmatism, age, and lens thickness
Kawahara 2021 [26]	Multivariate	0.96	0.72	Corneal and refractive astigmatism

WTR: with-the-rule, ATR: against-the-rule. * Upper row: coefficient of determination calculated when corneal astigmatism is measured by keratometer; lower row: value calculated when measured by Scheimpflug camera.

**Table 2:** Corneal astigmatism measurement and surgical technique employed in each study in Table 1.

Author	Measured Corneal Astigmatism	Corneal Astigmatism Measurement Equipment	Surgical Technique	
			Incision	IOL
Teus et al. 2010 [21]	Anterior	Keratometer	3.2 mm corneal incision (Side ports unknown)	3-piece aclylic
Tejedor et al. 2013 [22]	Anterior (keratometer) and total (Scheimpflug camera)	Keratometerand Scheimpflug camera	2.75 mm corneal incision (Side ports unknown)	1-piece aclylic
Leffler et al. 2008 [23]	Anterior	Keratometer	Scleral or corneal incision (Size and side ports unknown)	Unknown
Kawahara et al. 2017 [24]	Anterior	Keratometer	2.0 mm corneal incision and 1 side port	1-piece aclylic
Kawahara et al. 2020 [25]	Total	Scheimpflug camera	2.4 mm corneal incision and 2 side port	1-piece aclylic
Kawahara 2021 [26]	Total	Anterior segment OCT	2.4 mm corneal incision and 1 side port	1-piece aclylic

IOL: intraocular lens, OCT: optical coherence tomography.

## Data Availability

Not applicable.
